# Bioinspired nanofilament coatings for scale reduction on steel

**DOI:** 10.3762/bjnano.16.3

**Published:** 2025-01-09

**Authors:** Siad Dahir Ali, Mette Heidemann Rasmussen, Jacopo Catalano, Christian Husum Frederiksen, Tobias Weidner

**Affiliations:** 1 Department of Chemistry, Aarhus University, 8000 Aarhus C, Denmark; 2 Department of Biological and Chemical Engineering, Aarhus University, 8000 Aarhus C, Denmark; 3 Danish Offshore Technology Centre, Danish Technical University, 2800 Kongens Lyngby, Denmark

**Keywords:** bioinspired materials, calcium carbonate, offshore assets, stainless-steel coating, super-hydrophobicity

## Abstract

Scaling of steel surfaces, prevalent in various industrial applications, results in significant operational inefficiencies and maintenance costs. Inspired by the natural hydrophobicity of springtail (Collembola) skin, which employs micro- and nanostructures to repel water, we investigate the application of silicone nanofilaments (SNFs) as a coating on steel surfaces to mitigate scaling. Silicone nanofilaments, previously successful on polymers, textiles, and glass, are explored for their hydrophobic properties and stability on steel. Our study demonstrates the successful coating of stainless steel with SNFs, achieving super-hydrophobicity and resilience under high shear stress and explosion/decompression tests. Scaling experiments reveal a 75.5% reduction in calcium carbonate deposition on SNF-coated steel surfaces. This reduction is attributed to altered flow dynamics near the super-hydrophobic surface, inhibiting nucleation and growth of scale. Our findings highlight the potential of bioinspired SNF coatings to enhance the performance and longevity of steel surfaces in industrial environments.

## Introduction

**Figure 1:**
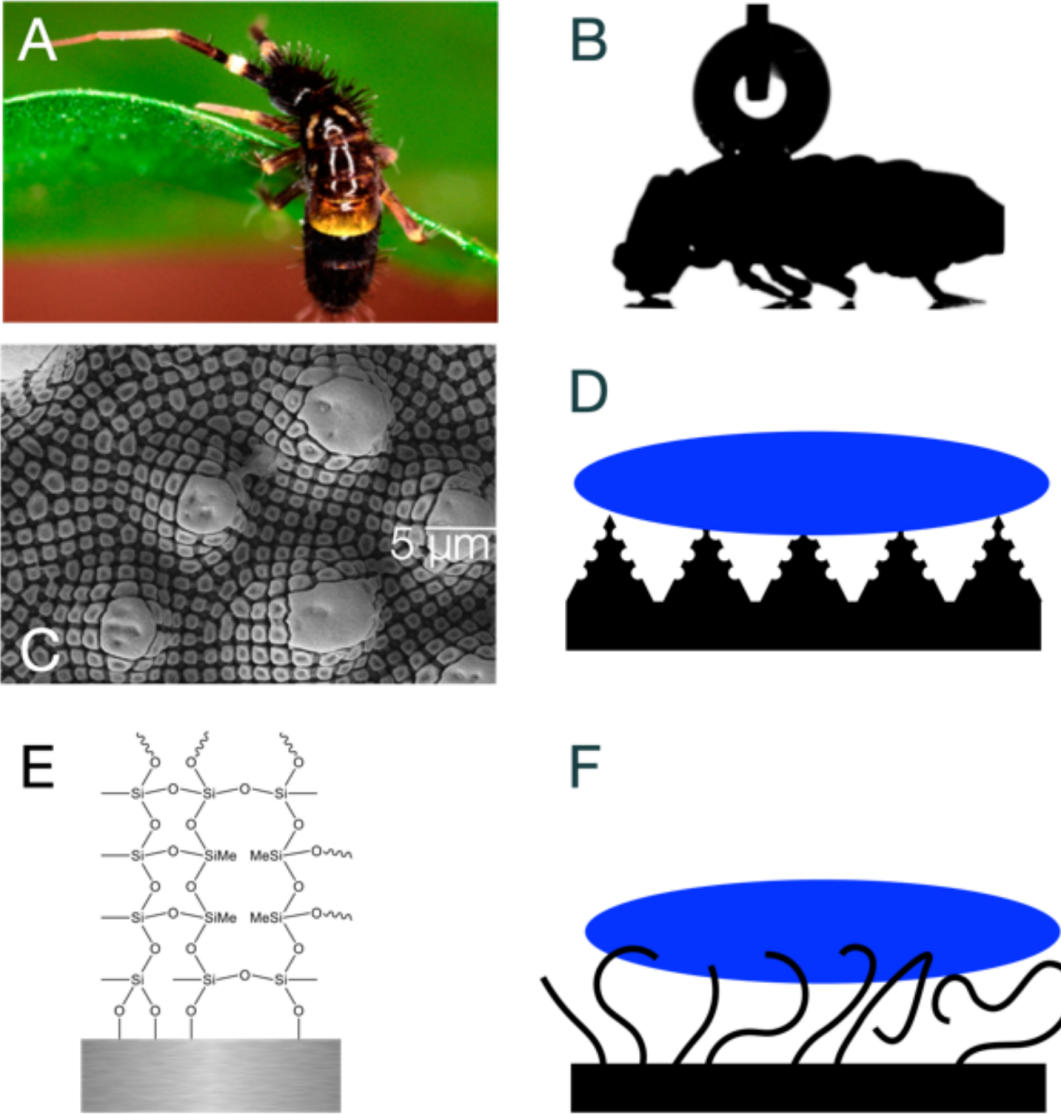
Bioinspired SNF coatings. (A) Springtails (Collembola) have micro- and nanostructured skin for effective water repellence. (B) High water contact angle on Collembola cuticle demonstrates natural super-hydrophobicity. (C,D) Multiscale structuring of the Collembola skin renders the surface hydrophobic [5]. (E) SNFs consist of silicone structure, which polymerize and grow on the surface. (F) SNF coating on steel mimics the multiscale springtail structuring. The coating decorates the surface with a water-repelling wire-like structure, which has been shown to be super-hydrophobic when bound to polymer, glass, and textiles. Figure 1A was adapted with permission from [5], Copyright 2020 American Chemical Society. This content is not subject to CC BY 4.0.

Small animals, such as insects, springtails (Collembola), and other hexapods, have distinctly large surface-to-volume ratios. This characteristic imposes significant challenges in terms of moisture control and water interaction [1–2]. The structure and chemistry of insect skin are finely tuned to navigate these challenges, showcasing a sophisticated natural adaptation to efficiently repel water [3]. Springtails have mastered this capability using micro- and nanostructured skin surfaces, which serve as a barrier against unwanted wetting [4–5]. Collembola breathe through their skin and, since they live in humid environments, need to retain air near their skin for survival in diverse habitats [6] (Figure 1A,B).

Drawing inspiration from Collembola, our study delves into the potential applications of mimicking the liquid-repelling properties of their skin to address a common industrial challenge: The scaling of steel surfaces. Scaling, a pervasive issue in various industries, results from unwanted water adhesion and mineral deposition, leading to corrosion, reduced efficiency, and increased maintenance costs. By understanding and replicating the nanostructured [3,7–9], liquid-repelling features of Collembola skin, we propose a novel approach to mitigate this issue. Figure 1 displays the cuticle micro- and nanostructure which leads to effective water repellency [5]. It has been shown how the multiscale structuring, from micron-sized hair-like structures all the way to nanometer-scale spikes and indentations can maintain the layer of air between the cuticle and water, which is needed for Collembola survival (Figure 1C,D).

Inspired by the intricate nanostructures found on Collembola skin, we fabricate silicone nanofilaments (SNFs) on steel surfaces. Here, the surface is coated by immersion of the surface in a solution of trichloromethylsilane (TCMS) in toluene in the presence of trace amounts of water (Figure 1E). This way, the surface is covered with a micrometer-thin layer of hard, stable SNFs. These filaments have been developed for coatings on polymers [10–12], textiles [12–17], aluminium and titanium [11–12], and glass [11–13,18–19], to replicate the water-repelling effects observed in nature [13,18,20–21]. Coatings with nano- or micrometer-sized protrusions that have an overhanging shape and low surface energy can effectively render a surface super-hydrophobic (Figure 1D,F). This texture creates an energy barrier, causing water droplets to rest on the protrusions while trapping air beneath them. In analogy with the texture of Collembola skin, this phenomenon, known as the Cassie state, results in the liquid being more in contact with air than with the solid surface, leading to high apparent contact angles [22]. The high contact angles also become apparent in Figure 1B, where a water droplet is resting on the Collembola surface.

Here, we explore the stability and hydrophobicity of these bioinspired nanofilaments on steel, a key material for industrial antiscaling applications that has not been investigated in this context. We test whether SNF coatings can prevent scaling of steel surfaces. In the following session, we describe a reliable procedure to coat stainless steel (Type 316) with nanofilaments, discuss stability test of the coatings, and the results of scaling experiments.

## Results and Discussion

### Nanofilamant performance and stability on steel

#### Shear stress test of SNF coatings on steel

Previous applications of SNF technology have been focused on materials with surface chemistries with polar functional groups and particularly hydroxy groups. To coat surfaces with a layer of SNFs, samples are immersed in a solution of trichloromethylsilane (TCMS) in toluene, in the presence of trace amounts of water (see Materials Section for details). After a reaction time of six hours, the surface is coated with a micrometer layer of SNFs. The surface reaction is proceeded by hydrolysis of TCMS due to water in the solvent. Subsequently, hydrolyzed TCMS molecules react with surface hydroxy moieties at the interface and thereby induce the polymerization of a polysiloxane on the material surface [18]. The polysiloxane methyl groups lower the surface energy and render the surface hydrophobic.

**Figure 2:**
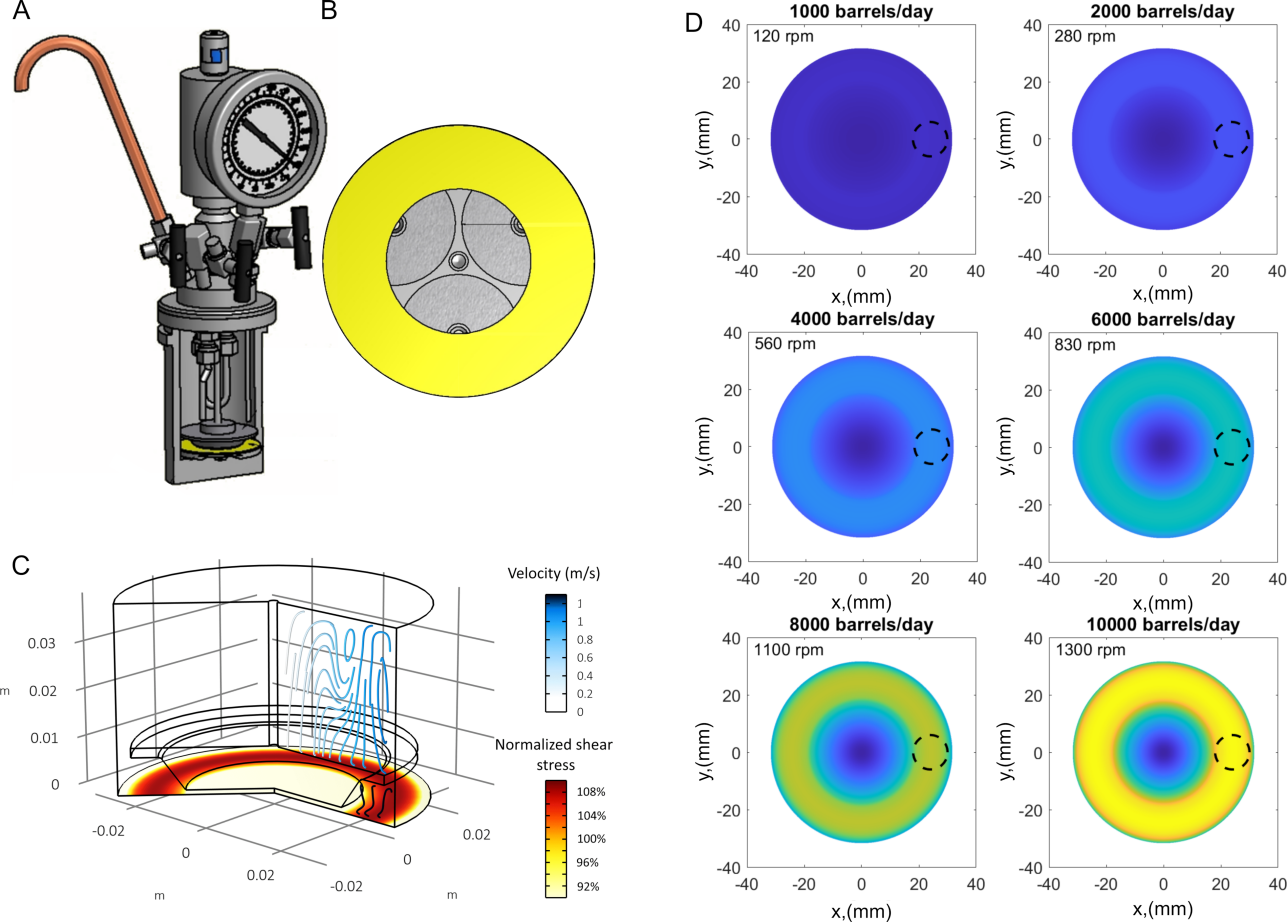
(A) Rendering of medium-pressure, medium-temperature, constant sheer stress device. Maximum operating temperature of 200 °C and maximum pressure of 200 bar. The liquid phase is pressurized via an inert gas (e.g., Ar or N_2_). (B) Top view of the static plate where three samples are mounted; around half of the surface area for each sample is exposed at constant sheer stress. (C) Steady state simulation of the velocity profile (reported as streamlines) and normalized sheer stress at the plate (contour plot). This latter parameter is normalized with respect to the sheer stress value calculated for a flow in a 4-inch nominal pipe at a volumetric flow of 10,000 barrels per day and 100 °C. (D) Contour plots of the shear stress at the plate for different rotational speeds and respective values of volumetric flows for a 4-inch pipe. The circled area represents the region of constant-sheer stress.

Clearly, the surface chemistry of steel surfaces is very different from the previously used materials such as glass [11–13,18], polymers [10–12], and textiles [11–16] as it does not offer the hydroxy moieties used previously for direct surface polymerization. It is therefore important to carefully test the stability and attachment of any SNFs growing on steel surfaces. Mechanical scratching of the coated steel surface will damage the coating visibly. Yet, most paintings and coatings will sustain damage when mechanically scratched. To evaluate shear stability under realistic operating conditions, we constructed a medium-temperature, medium-pressure, constant shear stress device (Figure 2A and Figure 2B). Our design is an adapted version of a cone-plate rheometer. Different from a classical design, we have iteratively optimized the design of the rotating truncated cone to provide a near-uniform shear stress at the static plate surface, where the SNF-coated samples are located (Figure 2C and Figure 2D). The tests of SNF coatings on steel were conducted under conditions relevant to oil production, which represents one of the most challenging applications for steel coatings. Specifically, the tests were performed in a 3 wt % toluene/water emulsion at a pressure of 70 bar and a temperature of 100 °C, with shear stresses at the plate corresponding to the flow of oil/water equivalent to 10,000 barrels per day in a 4-inch nominal steel pipe. Figure 2C shows a simulation of the fluid streamlines and shear stress on the samples matching the shear stress value in a 4-inch pipe at a flow rate of 10,000 barrels per day. The shear stress at the plate is reported in Figure 2D for different angular velocities and corresponding volumetric flow rates.

#### Super-hydrophobicity of SNF coatings on steel

**Figure 3:**
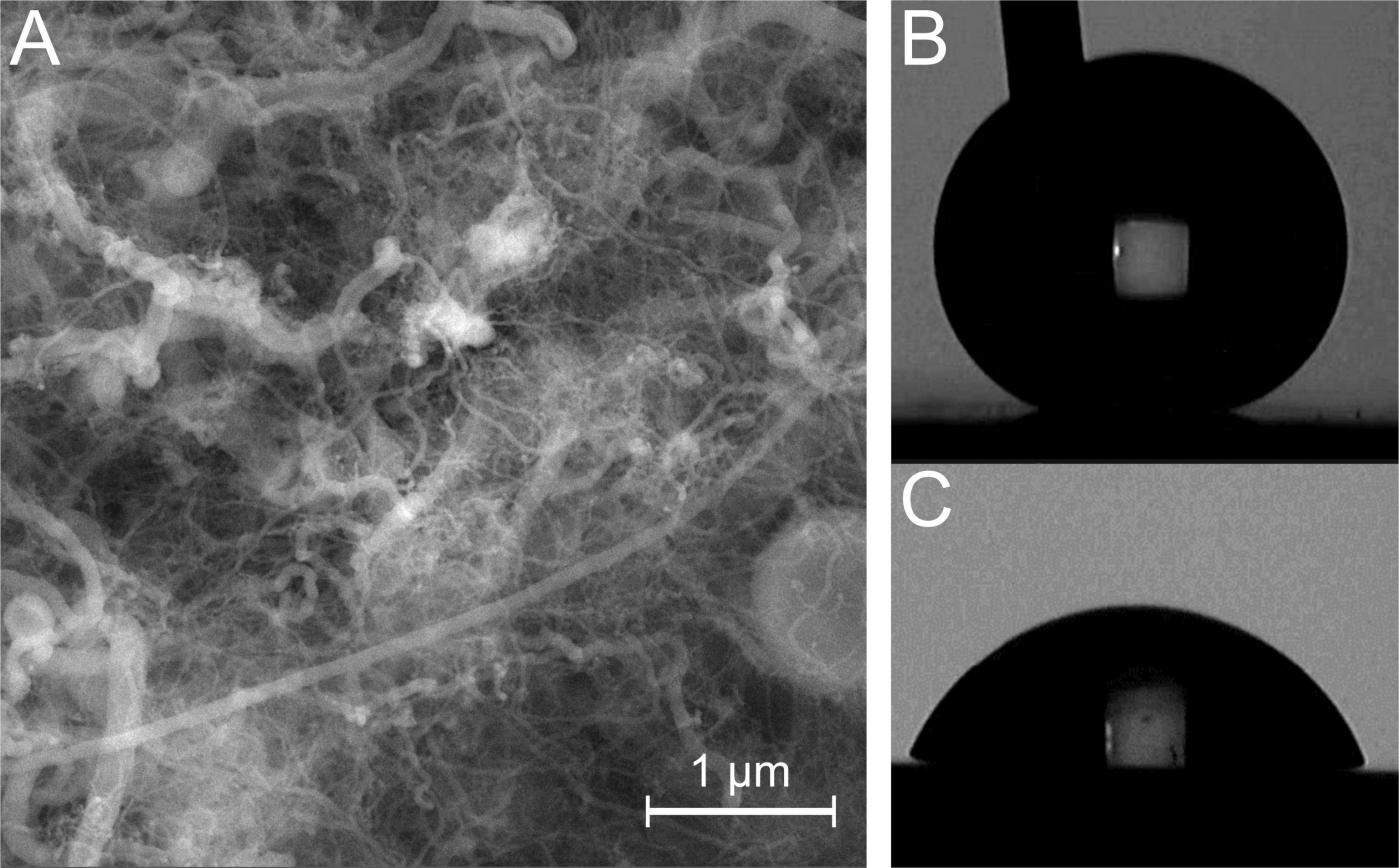
(A) SEM image of the coated surface. A network of SNFs with different width and diameters is visible. (B,C) Water contact angle measurements for the coated and uncoated samples.

**Figure 4:**
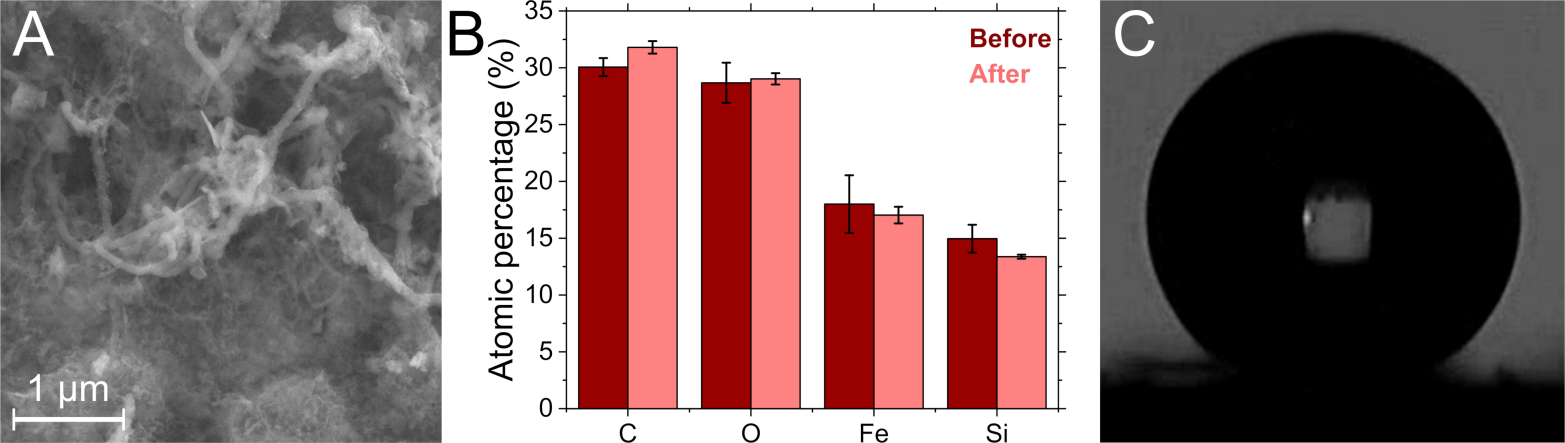
(A) SEM image of the SNF coating after the shear stress test. The SNF coating is still visible. (B) Composition of the SNF-coated steel before and after the shear stress test based on EDX analysis. (C) Image showing the contact angle measurement of the sample after the shear stress test. The coating is still water repelling.

Figure 3A shows a scanning electron microscopy (SEM) image of a stainless-steel surface coated with SNFs. The wire-type structures expected for SNF coating are clearly visible from a coated steel surface. Figure 3B and Figure 3C show the images of the measured angle of the coated and uncoated steel surfaces. The related atomic composition of the coating was analyzed by energy-dispersive X-ray spectroscopy (EDX) for both coated steel surfaces. The elemental composition in Figure 4B shows the silicon emission expected for the SNF film, which is not present on the bare steel surface. Oxygen and carbon, additional components of the SNF coating, are also detected for the SNFs. At the same time, these elements are also observed on the bare steel substrate and the elemental composition of the SNFs is difficult to disentangle.

**Figure 5:**
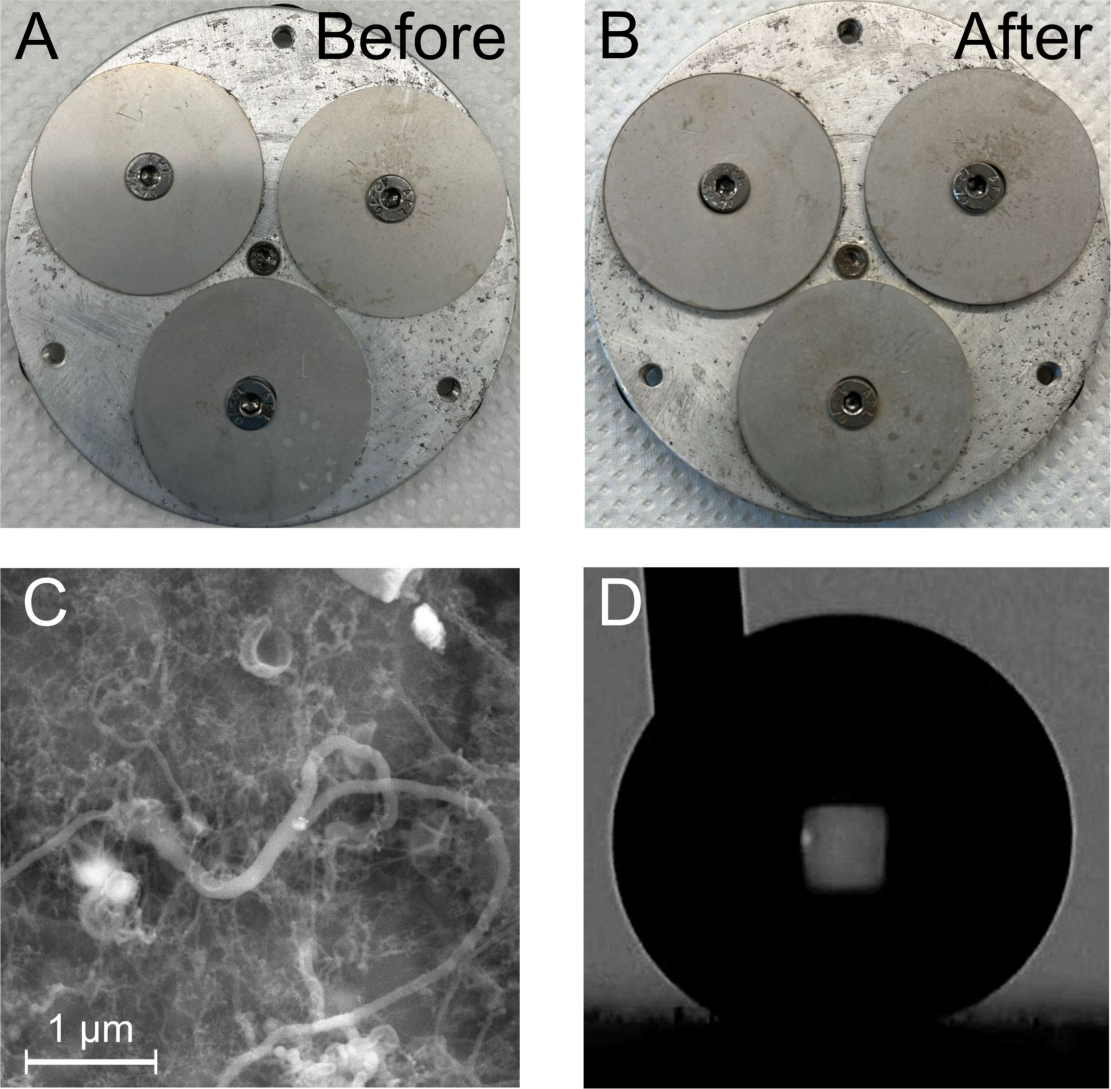
(A,B) Photos of the samples before and after the explosion/decompression test. The samples are still intact after the test, no delamination is visible on the samples. (C) SEM image of an SNF surface coating after the decompression/explosion test. No changes of the filament structure are discernible. (D) The water contact angle after the test shows that that coating is still functional after the test.

The contact angle of the uncoated surfaces was 71°. The contact angle of the SNF-coated surfaces is more difficult to measure as the droplet will not attach itself to the surface seen for Figure 3B and Figure 5D, in agreement with what has been reported before for super-hydrophobic SNF coatings on polymers [18], glass [23], and textiles [16]. Since the water contact is well above 150° and droplets are highly mobile (please see the videos in the Supporting Information File 1–3) – the definition of super-hydrophobicity – we can conclude that nanofilaments can be deposited on steel surfaces and that the grown structures render the steel surface super-hydrophobic [24].

The morphology and function of the SNF steel coatings remain intact during the shear stress test. The SEM image of the SNF coating after shear exposure in Figure 4A shows no damage of ablation of the structure. This is also supported by the EDX analysis, which shows that the composition is not changed within the error margins as the amount of oxygen and silicone remains constant in Figure 4B. The water contact angle (Figure 4C) is reduced a bit from the original value to 146 ± 3°, still at the limit to super-hydrophobicity.

To further test the coating stability, we also performed an explosion/decompression test after NACE TM0185, a standardized industry test of coating delamination. Here, the samples are pressurized in the autoclave and the pressure is quickly released after 24 hours at 100 °C and 100 bar. The pressure is reduced to 50 bar over a period of five minutes and afterwards the pressure is reduced to ambient pressure over a period of ten minutes. Any blisters or cracks in the coating indicate delamination or mechanical failure. As can be seen in in the optical images in Figure 5A and Figure 5B, no defects are visible after the explosion/decompression test, demonstrating the stability of the SNF coating on steel. This is also borne out by the SEM analysis (Figure 5C), which shows the SNF structures are fully intact after the test. The water contact angle also remains unchanged after the decompression/explosion test (Figure 5D).

### Scale reduction on SNF-coated steel surfaces

**Figure 6:**
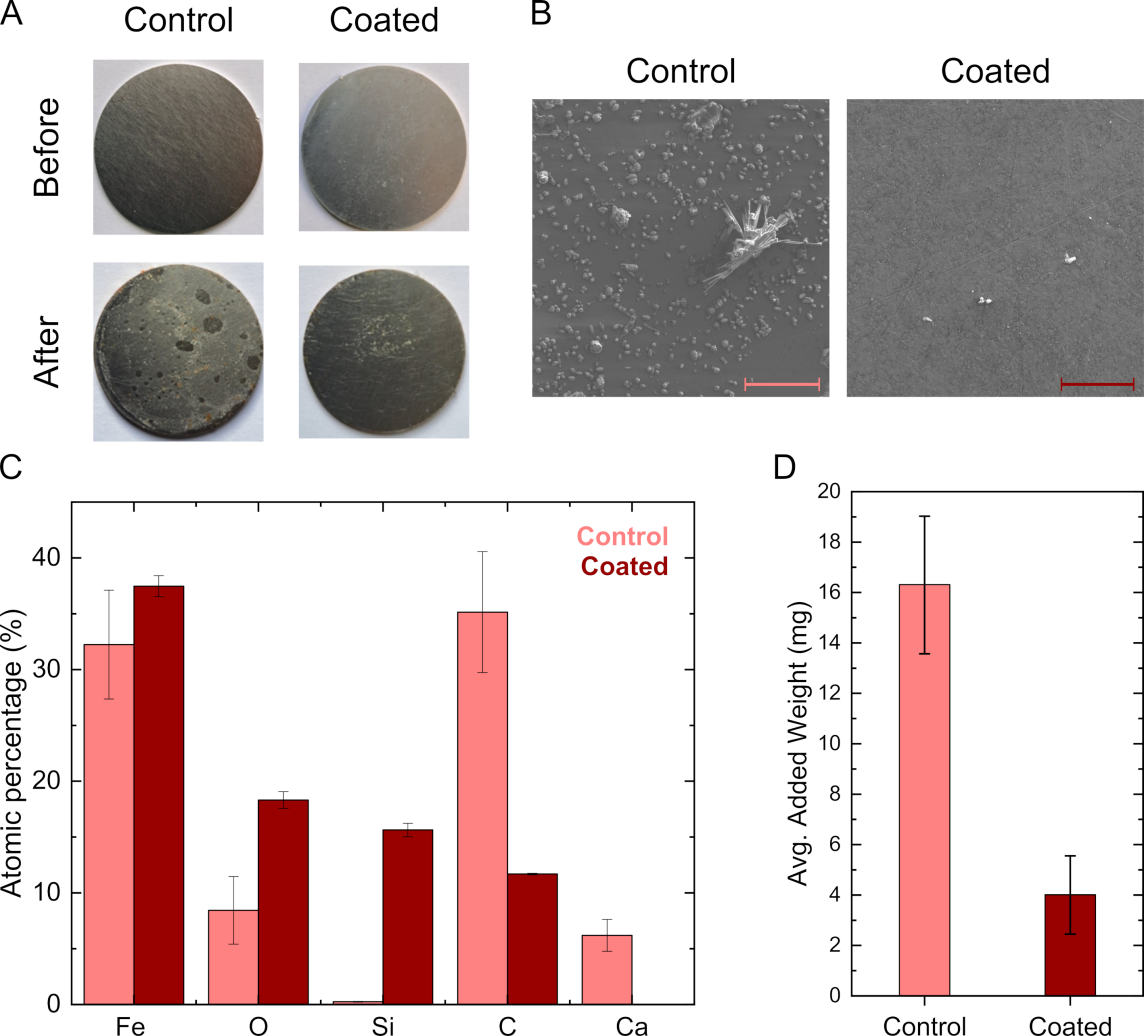
(A) Photos of the stainless-steel samples before and after experiments for the control and coated samples. (B) SEM images of the control and coated stainless-steel samples after the scale experiments. The scale bar depicts 200 µm. (C) Comparison of the relevant elements on the samples after the scale experiments measured with EDX. (D) Averaged added weight on the samples after the scale experiments.

The scaling of SNF samples was tested in a flow loop designed for the observation of calcium carbonate scaling at surfaces. Coated and uncoated steel surfaces where mounted in the sample holder within the test loop (see Methods Section for details) and exposed to a sequence of solutions. First, a solution of synthetic seawater and 3% texatherm oil was used to prime the samples. Then, a supersaturated solution of sodium carbonate and calcium chloride was added to the flow. After three hours, the samples were briefly rinsed with water in the flow loop, retrieved from the sample holder, and then dried in an oven at 80 °C. Figure 6A shows photos of coated and uncoated steel surfaces taken before and after exposure to the flow loop. While the uncoated sample shows significant scaling, small amounts of deposits are visible by the naked eye on the SNF-coated sample. This is also visible in the SEM images shown in Figure 6B. An EDX analysis of the scale clearly shows that the grown deposits mainly consist of calcium carbonate (Figure 6C). For a quantitative analysis of the extent of scaling, the samples were weighed before and after exposure to the scaling experiment. The results are summarized in Figure 6D. The gravimetric analysis shows that without the coating, the samples gained 16.3 ± 2.7 mg of calcium carbonate, while SNF-coated samples gained only 4.0 ± 1.5 mg. This corresponds to a scale reduction of 75.5% by the SNF coating.

Ostensibly, the non-wetting properties of the SNF films prevent attachment and growth of scale. Calcium carbonate scale formation starts with crystallization nuclei, small clusters of ions which can homogeneously form in the bulk solution or heterogeneously on material surface [25]. Once nucleation occurs, calcium carbonate crystals start to grow. The growth process involves the continuous deposition of Ca^2+^ and CO_3_^2−^ ions onto the surface-bound nuclei. Over time, these crystals increase in size and adhere more strongly to the steel surface and individual crystals coalesce to form larger, continuous scale layers. While it is unclear how, in detail, an SNF coating can affect the scale growth process, we hypothesize that the flow characteristics near the surface play an important role. While at regular solid–liquid interfaces, the flow velocity is assumed to be zero when modeling viscous drag, on super-hydrophobic surfaces the shear is reduced, leading to slip of the liquid across the surfaces and a non-zero flow velocity [26]. Flow at the material interface can hinder or prevent settling, nucleation, and growth of calcium carbonate scale at the interface.

## Conclusion

In conclusion, our study demonstrates the potential of biomimetic approaches to address the industrial challenge of scaling on steel surfaces. By drawing inspiration from the unique water-repelling properties of Collembola skin, we have fabricated silicone nanofilaments on steel surfaces, a novel application that has not been previously reported. The results indicate that SNF coatings can effectively render steel surfaces super-hydrophobic, as evidenced by high contact angles, high droplet mobility, and stable morphology under shear stress and explosion/decompression tests.

Furthermore, SNF-coated steel surfaces exhibited a marked reduction in calcium carbonate scaling compared to uncoated surfaces. This finding demonstrates the effectiveness of SNF coatings in mitigating scale formation, which is a significant challenge in the food industry, household appliances, and oil production. The structural integrity under harsh conditions further underpins the potential for industrial antiscaling solutions.

## Methods Section

### Coating procedure

The coating solution was prepared with 100 mL of toluene (108-88-3, VWR) with a water content between 250–300 ppm and mixing 0.8 mL trichloromethylsilane (75-79-6, Sigma-Aldrich) into the toluene. The water content was measured with a Karl-Fischer volumetric titration setup. The solvent used for the coating is prepared by mixing oversaturated toluene (>400 ppm) with toluene from the vendor (<200 ppm). The SNF coating is based on a dip coating, where the mixture is strongly stirred with the syringe for 40 seconds. The stainless-steel samples were quickly added to the chamber whose lid was sealed with Parafilm and the reaction was left to run for three hours. The procedure was repeated two times for a total of six hours of coating. For the results presented here, the water content in toluene was between 290–300 ppm. A coating with lower and higher amounts of water in the solvent is feasible, however, a less uniform coating will be obtained. Before the coating procedure the stainless-steel samples were cleaned by sonication for five minutes in toluene, acetone, and Milli-Q water, and the samples were rinsed with the solvent following in the sequence before each sonication step. Finally, the samples were dipped into ethanol and dried. The samples were washed in toluene, acetone and Milli-Q water, toluene, and dried after the first three hours of coating. When the coating was finished after a total of six hours, the samples were washed in toluene and left in Milli-Q water for approximately 12 hours. The last washing step ensures that all produced HCl is removed from the coating which will otherwise induce corrosion of the samples.

### Scanning electron microscopy and energy-dispersive X-ray spectroscopy measurements

Scanning electron microscopy images were acquired in a TESCAN CLARA (S8151) using the ANALYSIS and the UH-resolution scan mode with an accelerator voltage of 15 keV and 10 keV, a beam current of 300 pA, and a working distance of ≈10 mm and ≈7 mm in high-vacuum mode (pressure < 0.04 Pa). The SEM images were collected using an in-chamber Everhart−Thornley detector which identifies secondary electrons.

### Contact angle measurements

The measurements were performed by the sessile drop method using an FTA1000 contact angle system (First Ten Angstroms, Inc.) at ambient temperature. The volume of the water droplets was 2.5–3.0 µL.

### Autoclave stability and decompression/explosion tests

The autoclave (Parr series 5521) is based on a 300 mL vessel and reactor controller (4848, Parr). A home-build disc and sample holder was used. The sample holder is stationary and was placed at the bottom of the reactor chamber with the disc mounted on a spring to ensure that the distance between the disc and the samples was 1 mm. The shape of the disc is described in the text and gave a shear stress that mimics the condition offshore. The rotation of the disc was controlled by an RPM-meter, which was based on a teensy 3.6 microcontroller which scans a plate with 18 slots directly mounted on the reactor shaft. The signal was detected using library code and the RPM was subsequently calculated and shown on an LCB display.

The stability test experiments were run at 70 °C and 100 bar using pressurized argon and 700 RPM corresponding to 5000 bbl/day. The stability test has ben previously run at 300, 700, and 1300 RPM which corresponds to approx. 2000, 5000, and 10000 bbl/day, respectively. The disc and the sample holder were cleaned by rinsing in acetone, Milli-Q water, acetone, and afterwards left to air dry. The samples were placed in the sample holder and 100 mL of a mixture of 3 mL toluene and 97 mL Milli-Q water were added to the reaction chamber. The chamber was sealed and, after reaching the desired pressure and temperature, the experiment ran for three hours. The experiment was ended by releasing the pressure. The vessel was then placed in an ice water bath to lower the temperature to room temperature.

For the decompression/explosion test, the reaction chamber was cleaned by rinsing in acetone, Milli-Q water, acetone, and afterwards left to air dry. The samples were placed in the sample holder and a methane flask was attached to the reaction chamber and the pressure was set to 100 bar with a temperature of 100 °C. When the settings were reached, the experiment ran for 24 hours. The experiments commenced in two steps with the temperature kept at 100 °C. First, the pressure was reduced from 100 bar to 50 bar over a period of five minutes, and secondly the pressure was reduced from 50 bar to 1 bar over a period of ten minutes. Finally, the reaction chamber was placed in an ice water bath to lower the temperature to room temperature.

### Scale experiments

All samples were weighed and photographed prior to testing. The samples were then loaded onto the sample holder, and a solution of synthetic seawater and 3% oil was flushed through the sample holder to prime the samples for three minutes. Synthetic seawater (pH 6), containing calcium carbonate, was pumped from one tank while soda ash (pH 11), containing sodium carbonate, was pumped from another tank. The solution had pH 9, leading to the formation of calcium carbonate. The flow rate was maintained at 0.88 L/min and the experiment was conducted for three hours at room temperature. After three hours, the suction line was switched to demineralized water and the samples were rinsed for three minutes to remove all loose particles.

The sample holder with loaded samples was dried in an oven at 80 °C. The samples were weighed, and photos were taken afterwards.

## Supporting Information

The Supporting Information features three different contact angle videos.

File 1 Contact angle of the coated sample before the stability test.

File 2 Contact angle of the coated sample after the stability test.

File 3 Contact angle of the coated sample after the decompression/explosion test.

## Data Availability

The data that supports the findings of this study is available from the corresponding author upon reasonable request.
